# Simplexins P–S, Eunicellin-Based Diterpenes from the Soft Coral *Klyxum simplex*

**DOI:** 10.3390/md10061203

**Published:** 2012-05-25

**Authors:** Shwu-Li Wu, Jui-Hsin Su, Chiung-Yao Huang, Chi-Jen Tai, Ping-Jyun Sung, Chih-Chung Liaw, Jyh-Horng Sheu

**Affiliations:** 1 Department of Marine Biotechnology and Resources, National Sun Yat-sen University, Kaohsiung 804, Taiwan; Email: wusl@webmail.nkmu.edu.tw (S.-L.W.); betty8575@yahoo.com.tw (C.-Y.H.); jean801023@hotmail.com (C.-J.T.); ccliaw@mail.nsysu.edu.tw (C.-C.L.); 2 Center of General Studies, National Kaohsiung Marine University, Kaohsiung 811, Taiwan; 3 National Museum of Marine Biology & Aquarium, Pingtung 944, Taiwan; Email: x2219@nmmba.gov.tw (J.-H.S.); pjsung@nmmba.gov.tw (P.-J.S.); 4 Graduate Institute of Marine Biotechnology, National Dong Hwa University, Pingtung 944, Taiwan; 5 Division of Marine Biotechnology, Asia-Pacific Ocean Research Center, National Sun Yat-sen University, Kaohsiung 804, Taiwan

**Keywords:** soft coral, eunicellin-based diterpenes, cytotoxicity, *Klyxum simplex*

## Abstract

Four new eunicellin-based diterpenes, simplexins P–S (**1–4**), and the known compound simplexin A (**5**), have been isolated from the soft coral *Klyxum simplex*. The structures of the new metabolites were determined on the basis of extensive spectroscopic analysis, particularly 1D and 2D NMR experiments. Compounds **1 **and **3–5** were shown to exhibit cytotoxicity against a limited panel of cancer cell lines, **3** being the most cytotoxic.

## 1. Introduction

**Chart 1:**
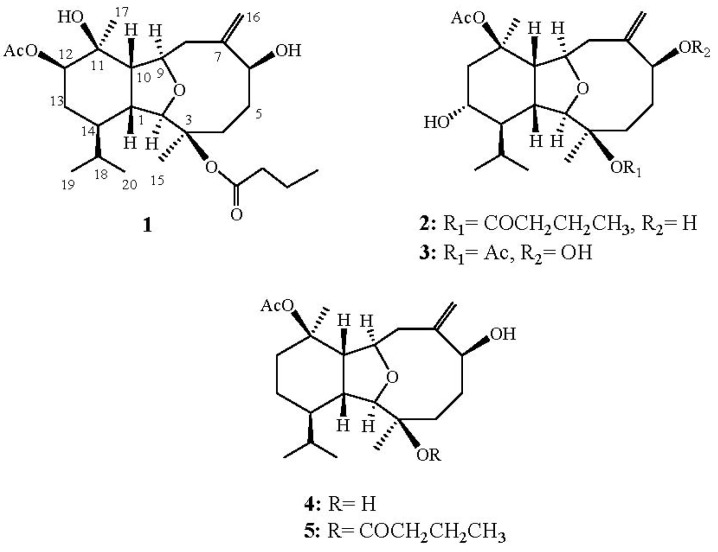
Structures of Metabolites **1–5**.

In the investigation of the bioactive metabolites from soft corals of Taiwanese waters, many bioactive eunicellin-based diterpenoids have been isolated from wild-type octocoral *Pachyclavularia violacea* [1,2], *Cladiella australis* [3], *Vigularia juncea* [4], *Cladiella hirsute* [5], *Cladiella krempfi* [6], *Klyxum molle* [7], and a cultured soft coral *Klyxum simplex* [8,9,10,11]. Our previous study on the secondary metabolites of a Dongsha Atoll soft coral *K. simplex* Thomson & Dean (Alcyonacea, Alcyoniidae) has resulted in the isolation of a series of new eunicellin-based diterpenoids, simplexins A–O [12,13]. In continuation of our search for metabolites from the Dongsha Atoll soft coral *K. simplex*, we have isolated another four new eunicellin-type metabolites, simplexins P–S (**1–4**) (Chart 1) and a known compound simplexin A (**5**). The structures of **1**–**4** were established by extensive spectroscopic analysis, including careful examination of 2D NMR (^1^H–^1^H COSY, HMQC, HMBC and NOESY) correlations. The cytotoxicity of **1–5** against human erythroleukemia (K562), human leukemia (CCRF-CEM), human breast earcinoma (T47D), and human lymphoid T (MOLT 4) cell lines was investigated. The results showed that compound **3**, being the most cytotoxic, is worthy of further biomedical investigation.

## 2. Results and Discussion

**Figure 1:**
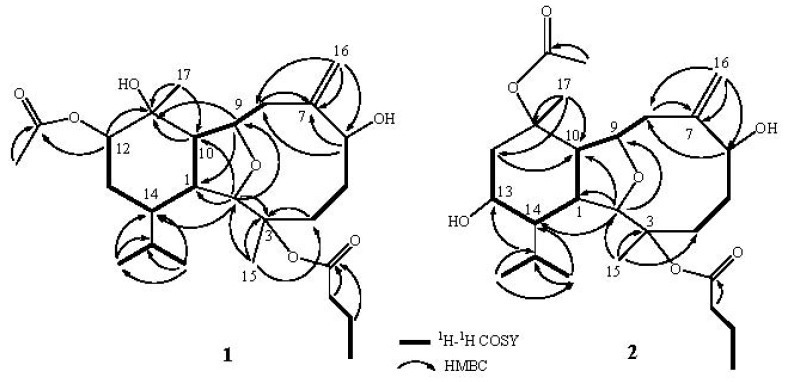
Key ^1^H–^1^H COSY and HMBC correlations of **1** and **2**.

Simplexin P (**1**) was obtained as a white powder. Its molecular formula C_26_H_42_O_7_ was determined by the HRESIMS (*m/z* 489.2827 [M + Na]^+^) was which deduced six degrees of unsaturation. The IR absorptions bands at ν_max_ 3255 and 1717 cm^–^^1^ revealed the presence of hydroxy and ester carbonyl functionalities. The ^13^C NMR spectrum measured in CDCl_3 _showed signals of 26 carbons (Table 1) which were assigned as six methyls, six sp^3^ methylenes, one sp^2^ methylene, eight sp^3^ methines (including four oxymethines), two sp^3^ and three sp^2^ quaternary carbons (including two ester carbonyls) by DEPT. In the ^13^C NMR spectrum of **1**, two carbonyl resonances at δ 172.6 and 170.2 ppm confirmed the presence of two ester groups. In the ^1^H NMR spectrum of **1** (Table 2), one acetate methyl (δ 2.12) and one *n-*butyryloxy [δ 0.92 (3H, t, *J* = 7.5 Hz), 1.60 (2H, m), and 2.13 (2H, m)] groups were observed. Moreover, two ^1^H NMR singlet signals at δ 5.13 and 5.46 revealed the presence of one olefinic methylene. In addition, the diagnostic signals at δ 4.17 and 3.58 implied the presence of an ether linkage between C-9 and C-2. On the basis of the above results and by the assistance of ^1^H–^1^H COSY and HMBC experiments (Figure 1), the molecular framework of **1** could be established as an eunicellin-type skeleton. Furthermore, the acetoxy group positioned at C-12 was confirmed by HMBC correlations from oxymethine [δ 4.89 (H-12)] and acetate methyl (δ 2.12) to the ester carbonyl carbon at δ 170.2 (C). Thus, the remaining one *n*-butyryloxy group was located at C-3, an oxygen-bearing quaternary carbon resonating at δ 84.4 ppm. On the basis of the above analysis, the planar structure of **1** was established unambiguously. 

**Table 1:** ^13^C NMR data for compounds **1**–**4***^a^*.

Position	1	2	3	4
1	41.7 (CH) *^b^*	43.0 (CH)	43.1 (CH)	41.5 (CH)
2	89.8 (CH)	91.6 (CH)	91.4 (CH)	91.4 (CH)
3	84.4 (C)	84.5 (C)	84.6 (C)	74.1 (C)
4	28.7 (CH_2_)	29.4 (CH_2_)	30.0 (CH_2_)	35.2 (CH_2_)
5	35.3 (CH_2_)	35.4 (CH_2_)	30.0 (CH_2_)	35.0 (CH_2_)
6	73.0 (CH)	73.5 (CH)	87.3 (CH)	74.1 (CH)
7	150.3 (C)	150.3 (C)	145.6 (C)	152.0 (C)
8	41.0 (CH_2_)	41.2 (CH_2_)	41.9 (CH_2_)	41.5 (CH_2_)
9	78.4 (CH)	79.2 (CH)	78.9 (CH)	78.2 (CH)
10	50.2 (CH)	45.5 (CH)	45.0 (CH)	46.4 (CH)
11	71.1 (C)	83.5 (C)	83.3 (C)	82.1 (C)
12	75.5 (CH)	42.2 (CH_2_)	42.7 (CH_2_)	32.4 (CH_2_)
13	24.2 (CH_2_)	66.8 (CH)	66.8 (CH)	18.1 (CH_2_)
14	43.4 (CH)	50.3 (CH)	49.8 (CH)	42.7 (CH)
15	22.2 (CH_3_)	22.7 (CH_3_)	22.9 (CH_3_)	27.6 (CH_3_)
16	116.9 (CH_2_)	117.0 (CH)	118.2 (CH)	117.2 (CH_2_)
17	26.2 (CH_3_)	25.2 (CH_3_)	25.3 (CH_3_)	25.4 (CH_3_)
18	27.4 (CH)	28.4 (CH)	28.5 (CH)	28.0 (CH)
19	21.7 (CH_3_)	24.8 (CH_3_)	24.8 (CH_3_)	21.8 (CH_3_)
20	15.5 (CH_3_)	15.8 (CH_3_)	15.7 (CH_3_)	15.0 (CH_3_)
3-Ac			22.3 (CH_3_)	
			169.9 (C)	
11-Ac		22.4 (CH_3_)	22.4 (CH_3_)	22.6 (CH_3_)
		170.0 (C)	170.0 (C)	170.3 (C)
12-Ac	21.2 (CH_3_)			
	170.2 (C)			
3- *n*-Butyrate	13.6 (CH_3_)	13.6 (CH_3_)		
	18.4 (CH_2_)	18.6 (CH_2_)		
	37.3 (CH_2_)	37.4 (CH_2_)		
	172.6 (C)	172.6 (C)		

*^a^* Spectra recorded at 125 MHz in CDCl_3_ at 25 °C; *^b^* Attached protons were determined by DEPT experiments.

**Table 2:** ^1^H NMR Data for compounds **1**–**4***^a^*.

Position	1	2	3	4
1	2.42 dd (12.0, 7.5) *^b^*	2.24 dd (11.5, 7.0)	2.20 dd (12.5, 7.0)	2.27 dd (11.5, 7.5)
2	3.58 s	3.59 s	3.58 s	3.56 s
4	2.20 m 1.80 m	2.17 m 1.84 m	2.10 m 1.97 m	1.73 m
5	2.12 m 1.71 m	2.10 m 1.73 m	2.13 m 1.54 m	2.06 m 1.95 m
6	4.33 dd (11.0, 4.0)	4.30 *br* d (10.5)	4.62 dd (10.5, 2.0)	4.32 *br* d (10.5)
8	2.84 dd (14.0, 4.5)	2.83 dd (14.0, 5.0)	2.83 dd (13.5, 5.0)	2.86 dd (13.5, 5.5)
	2.47 d (14.0)	2.47 d (14.0)	2.55 d (13.5)	2.51 d (13.5)
9	4.17 dd (11.0, 4.0)	4.13 dd (10.5, 4.5)	4.11dd (11.0, 5.0)	4.09 dd (10.0, 5.5)
10	2.66 dd (11.0, 7.5)	3.08 dd (10.5,7.5)	3.17 dd (10.5, 7.0)	2.96 dd (10.0, 7.5)
12	4.89 dd (11.7, 4.2)	1.52 m	1.54 m	1.44 m
		2.41 m	2.34 dd (13.5, 3.5)	2.25 m
13	1.61 m 1.70 m	3.90 ddd (15.0, 13.2, 4.5)	3.90 ddd (16.0, 11.0, 5.0)	1.34 m
				1.46 m
14	1.41 m	1.26 m	1.26 t (11.0)	1.19 m
15	1.59 s	1.56 s	1.53 s	1.16 s
16	5.13 s 5.46 s	5.22 s 5.47 s	5.35 s 5.46 s	5.30 s 5.61 s
17	1.21 s	1.57 s	1.58 s	1.54 s
18	1.92 m	1.92 m	1.89 m	1.79 m
19	0.95 d (7.0)	1.18 d (7.0)	1.19 d (7.0)	0.94 d (7.0)
20	0.83 d (7.0)	0.96 d (7.0)	0.97 d (7.0)	0.78 d (7.0)
3-acetate			1.95 s	
11-acetate		2.00 s	2.01 s	2.01 s
12-acetate	2.12 s			
3-*n*-butyrate	0.92 t (7.5)	0.94 t (7.5)		
	1.60 m	1.59 m		
	2.13 m	2.15 m		
6-OOH			7.78 s	

*^a^* Spectra recorded at 500 MHz in CDCl_3_ at 25 °C; *^b^* *J* values are (in Hz) in parentheses.

**Figure 2:**
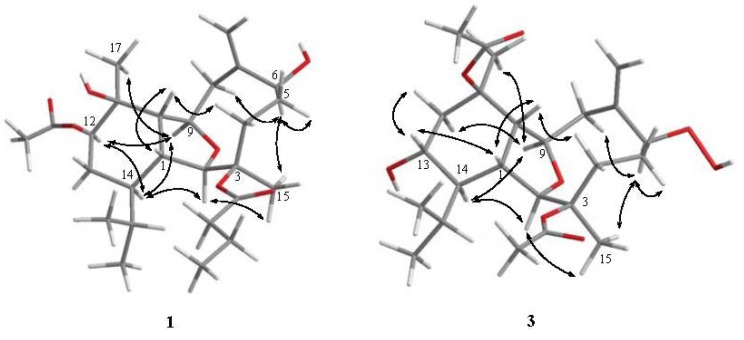
Selective NOESY correlations of **1** and **3**.

The relative configuration of **1** was determined by analysis of NOE correlations observed in the NOESY spectrum (Figure 2), which showed NOE interactions between H-1 and H-10, revealing they were both β-oriented. Also, correlations between H-2 and both H_3_-15 and H-14; H-14 and both H-9 and H-12; H-9 and both H-12 and H_3_-17; and H_3_-15 and H-6 suggested that of all of H-2, H-6, H-9, H-12, H-14, H_3_-15 and H_3_-17 were all α-oriented. Thus, the NOESY spectrum indicated that **1** was found to possess the (1R*, 2R*, 3R*, 6S*, 9R*, 10S*, 11S*, 12R*, 14R*)-configuration.

Compound **2**, simplexin Q, was assigned as the molecular formula C_26_H_42_O_7_ from its HRESIMS data, appropriate for six degrees of unsaturation. ^1^H and ^13^C NMR spectral data of **2** (Table 1 and Table 2) also showed the presence of one acetoxy group (δ_C_ 170.0, C; 22.4, CH_3_; δ_H_ 2.00, 3H, s) and one *n*-butyryloxy group (δ_C_ 172.6, C; 37.4, CH_2_; 18.6, CH_2_; 13.6, CH_3_; δ_H_ 2.15, 2H, m; 1.59, 2H, m; 0.94, 3H, t, *J* = 7.5 Hz). Comparison of the NMR data of **2** with those of the known compound simplexin A (**5**) [12], revealed that the only difference was the presence of an oxymethine (δ_H_ 3.90; δ_C_ 66.8) at C-13 in **2**, instead of the methylene (δ_H_ 2.27 and 1.44; δ_C_ 18.1) in **5** arising from the substitution of a hydroxy moiety at C-13 in **2**, instead of a methylene moiety at the same carbon in **1**. Furthermore, the molecular framework was also established by ^1^H–^1^H COSY and HMBC experiments (Figure 1). The relative configuration of **2,** deduced using a NOESY spectrum, is similar to that of **5**. In addition, H-13 was found to exhibit a NOE correlation with H-1 but not with H-14, revealing the α-orientation of the hydroxyl group at C-13. Therefore, the structure of **2** was found to possess the (1R*, 2R*, 3R*, 6S*, 9R*, 10S*, 11R*, 13R*, 14R*)-configuration.

Simplexin R (**3**), isolated as a white powder, was assigned a molecular formula C_24_H_38_O_8_ from high resolution ESIMS analysis. The presence of the acetate groups was indicated by IR absorption at 1733 cm^–1^, ^1^H NMR signals (Table 2) at δ 1.95 (s, 3H) and 2.01 (s, 3H) and ^13^C NMR (Table 1) signals at δ 22.3 (CH_3_), 22.4 (CH_3_), 169.9 (C) and 170.0 (C). The NMR spectral data of **3 **showed the presence of a 1,1-disubstituted carbon–carbon double bond (δ_C_ 118.2, CH_2_ and 145.6, C; δ_H_ 5.35, s and 5.46, s) and a hydroperoxy proton (δ_H_ 7.78, s). Comparison of the NMR data of **3** with those of **2 **revealed the replacement of the *n*-butyryloxy moiety at C-3 and the hydroxy group at C-6 in **2 **by the acetoxy and the hydroperoxy groups in **3**, respectively. The relative configuration of **3 **was determined mainly by the assistance of the NOESY experiment. The NOE correlations of **3** indicated that **3** possessed the same configurations for each chiral center as those of **2**.

Simplexin S (**4**) showed the pseudomolecular ion peak [M + Na]^+^ at*m/z* 403.2463 in the HRESIMS and the molecular formula was determined as C_22_H_36_O_5_. NMR spectroscopic data of **4** (Table 1 and Table 2) showed the presence of one acetoxy group (δ_C_ 170.3, C; 22.6, CH_3_; δ_H_ 2.01, 3H, s). Comparison of the NMR data of **4** with those of **5 **revealed that the only difference between both compounds arises from the replacement of the hydroxy group at C-3 in **4 **by one *n*-butyryloxy moiety in **5**. The NOESY spectrum indicated that **4** was found to possess the (1R*, 2R*, 3R*, 6S*, 9R*, 10S*, 11R*, 14R*)-configuration.

The cytotoxicity of compounds **1**–**5** against the proliferation of a limited panel of cancer cell lines, including K562, CCRF-CEM, T47D, and MOLT 4 was evaluated by the Alamar Blue assay, using 5-fluorouracil as a positive control. It was found that **3** showed activity against the proliferation of K-562, CCRF-CEM, T47D, and MOLT 4 cancer cells (ED_50_ values of 7.2 ± 2.4, 2.7 ± 0.1, 13.5 ± 2.8, and 3.8 ± 0.5 µg/mL, respectively) (Table 3).

**Table 3:** Cytotoxicities of Compounds **1**–**5**.

	Cell Lines ED_50_ (µg/mL)			
Compound	K-562	CCRF-CEM	T47D	MOLT 4
**1**	>20	12.0 ± 1.6	>20	30.3 ± 3.4
**2**	>20	>20	>20	>20
**3**	7.2 ± 2.4	2.7 ± 0.1	13.5 ± 2.8	3.8 ± 0.5
**4**	>20	13.0 ± 0.9	>20	16.4 ± 3.1
**5**	>20	17.0 ± 2.9	>20	18.2 ± 2.6
5-Fluorouracil	2.3 ± 0.2	1.8 ± 0.3	9.8 ± 1.5	2.3 ± 0.3

## 3. Experimental Section

### 3.1. General Experimental Procedures

Melting points were determined using a Fisher-Johns melting point apparatus and were uncorrected. Optical rotations were measured on a JASCO DIP-1000 digital polarimeter. IR spectra were recorded on a JASCO FT/IR-4100 infrared spectrophotometer. ESIMS were obtained with a Bruker APEX II mass spectrometer. NMR spectra were recorded on a Varian Unity INOVA 500 FT-NMR at 500 MHz for^ 1^H and 125 MHz for ^13^C in CDCl_3_. Si gel 60 (Merck, 230–400 mesh) was used for column chromatography. Precoated silica gel plates (Merck, Kieselgel 60 F_254_, 0.2 mm) were used for analytical TLC. High-performance liquid chromatography was performed on a Hitachi L-7100 HPLC apparatus with a Merck Hibar Si-60 column (250 × 21 mm, 7 µm).

### 3.2. Animal Material

*Klyxum simplex* (230 g, wet wt), was collected by hand using scuba off the coast of Dongsha Atoll, in September, 2006, at a depth of 11 m, and stored in a freezer until extraction. A voucher sample (specimen No. 20060901-1) was deposited at the Department of Marine Biotechnology and Resources, National Sun Yat-sen University.

### 3.3. Extraction and Separation

The frozen bodies of *K. simplex* (230 g, wet wt) were minced and exhaustively extracted with EtOAc (1 L × 4). The organic extract was evaporated under reduced pressure to give a residue (2.5 g) which was subjected to Si gel column chromatography and eluted with EtOAc in *n*-hexane (0–100%, gradient) to yield 22 fractions. Fractions 10–12 (1.05 g) eluted with EtOAc–*n*-hexane (1:3), were further purified over silica gel using EtOAc–*n*-hexane (1:3 to 1:1) to afford 46 subfractions. Subfraction 37 was also purified by normal phase HPLC using acetone–*n*-hexane (1:2) to afford **3 **(0.9 mg, 0.036%). Fractions 13–15 (0.47 g), eluted with EtOAc–*n*-hexane (1:1), were further purified over silica gel using EtOAc–*n*-hexane (1:1) to afford 19 subfractions. Subfraction 17 was separated by normal phase HPLC using acetone–*n*-hexane (1:2) to yield **4** (1.6 mg, 0.064%) whilst subfraction 19 was purified by normal phase HPLC using acetone–*n*-hexane (1:2) to afford **2** (1.3 mg, 0.052%). Fractions 16–19 (0.51 g) eluted with EtOAc–*n*-hexane (2:1), were further purified over silica gel using EtOAc–*n*-hexane (2:1) to afford 4 subfractions. Subfraction 4 was separated by normal phase HPLC using MeOH–CH_2_Cl_2_ (1:30) to afford **1** (3.3 mg, 0.132%).

Simplexin P (**1**): white powder (3.3 mg); mp 179.0–180.0 °C; [*α*]*^26^_D_* = −27 (*c* 1.2, CHCl_3_); IR (neat) ν_max_ 3255 (broad) and 1717 cm^–^^1^; ^1^H and ^13^C NMR data, see Table 1 and Table 2; ESIMS *m*/*z* 489 (100, [M + Na]^+^); HRESIMS *m*/*z* 489.2827 (calcd for C_26_H_42_O_7_Na, 489.2828).

Simplexin Q (**2**): colorless oil (1.3 mg); [*α*]*^26^_D_* = −11 (*c* 0.8, CHCl_3_); IR (neat) ν_max_ 3410 (broad) and 1732 cm^–^^1^; ^1^H and ^13^C NMR data, see Table 1 and Table 2; ESIMS *m*/*z* 489 (100, [M + Na]^+^); HRESIMS *m*/*z* 489.2830 (calcd for C_2__6_H_4__2_O_7_Na, 489.2828).

Simplexin R (**3**): white powder (0.9 mg); mp 167–168 °C; [*α*]*^26^_D_* = −27 (*c* 0.4, CHCl_3_); IR (neat) ν_max_ 3395 (broad) and 1733 cm^–^^1^; ^1^H and ^13^C NMR data, see Table 1 and Table 2; ESIMS *m*/*z* 477 (100, [M + Na]^+^); HRESIMS *m*/*z* 477.2467 (calcd for C_24_H_38_O_8_Na, 477.2464).

Simplexin S (**4**): colorless oil (1.6 mg); [*α*]*^26^_D_* = −41 (*c* 0.6, CHCl_3_); IR (neat, CHCl_3_) ν_max_ 3354 (broad) and 1716 cm^–^^1^; ^1^H and ^13^C NMR data, see Table 1 and Table 2; ESIMS *m*/*z* 403 (100, [M + Na]^+^); HRESIMS *m*/*z* 403.2463 (calcd for C_22_H_36_O_5_Na, 403.2460).

### 3.4. Cytotoxicity Testing

Cell lines were purchased from the American Type Culture Collection (ATCC). Cytotoxicity assays of compounds **1**–**5** were performed using the Alamar Blue assay [14,15].

In previous studies, a series of new eunicellin-based diterpenoids were isolated from the cultured and wild-type soft corals *Klyxum simplex*. Our continued investigation on the chemical constituents of wild-type soft coral *K. simplex* has again led to the isolation of four new eunicellin-based diterpenoids, simplexins P–S. Simplexin R (3) exhibited significant cytotoxicity against CCRF-CEM and MOLT 4 cells, and moderate to weak cytotoxicity against K-562 and T47D cells. Also, compounds **1**, **4** and **5** exhibited moderate to weak cytotoxicity toward CCRF-CEM and MOLT 4 cell lines. Besides our research, many recent studies have showed the versatile structures and bioactivities of eunicellin-type compounds [16,17,18,19,20,21]. These studies along with the new simplexins described here suggest that eunicellin-type compounds, in particular 3, are worthy of further biomedical investigation.

## Supplementary Files

Supplementary File 1: PDF-Document (PDF, 1673 KB)
