# Correction: Wu, S.-L., *et al.* Simplexins P–S, Eunicellin-Based Diterpenes from the Soft Coral *Klyxum simplex*. *Mar. Drugs* 2012, *10*, 1203–1211

**DOI:** 10.3390/md11125087

**Published:** 2013-12-13

**Authors:** Shwu-Li Wu, Jui-Hsin Su, Chiung-Yao Huang, Chi-Jen Tai, Ping-Jyun Sung, Chih-Chung Liaw, Jyh-Horng Sheu

**Affiliations:** 1 Department of Marine Biotechnology and Resources, National Sun Yat-sen University, Kaohsiung 804, Taiwan; E-Mails: wusl@webmail.nkmu.edu.tw (S.-L.W.); betty8575@yahoo.com.tw (C.-Y.H.); jean801023@hotmail.com (C.-J.T.); ccliaw@mail.nsysu.edu.tw (C.-C.L.); 2 Center of General Studies, National Kaohsiung Marine University, Kaohsiung 811, Taiwan; 3 National Museum of Marine Biology & Aquarium, Pingtung 944, Taiwan; E-Mails: x2219@nmmba.gov.tw (J.-H.S.); pjsung@nmmba.gov.tw (P.-J.S.); 4 Graduate Institute of Marine Biotechnology, National Dong Hwa University, Pingtung 944, Taiwan; 5 Division of Marine Biotechnology, Asia-Pacific Ocean Research Center, National Sun Yat-sen University, Kaohsiung 804, Taiwan

We found some errors in our previous published paper [1]. The structure of simplexin Q was found to be the same as klysimplexin C, previously published in *Tetrahedron* **2009**, *65*, 7016–7022 [2]. Also, simplexin S and a known compound cladieunicellin G, reported in *Chem*. *Pharm*. *Bull*. **2012**, *60*, 160–163 [3], have the same structure (see Figure 1). We apologize for any inconvenience caused to the readers by these errors.

**Figure 1:**
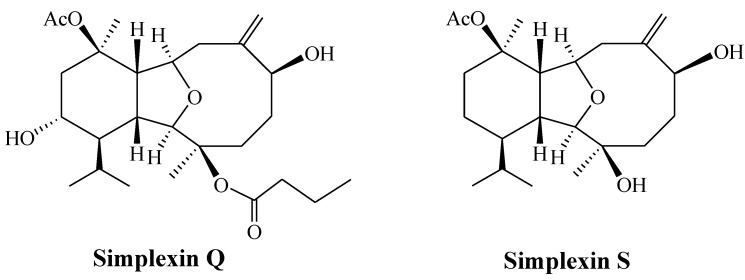
Chemical structures of simplexin Q and simplexin S.
