# Supplementary material for: Simplexins P–S, Eunicellin-Based Diterpenes from the Soft Coral Klyxum simplex
Source: Mar Drugs. 2012 May 25;10(6):1203–11. doi: 10.3390/md10061203 (PMC3397434; doi:10.3390/md10061203)
Supplement: Supplementary File 1: — PDF-Document (PDF, 1673 KB) [file marinedrugs-10-01203-s001.pdf]

Simplexins P–S, Eunicellin-Based Diterpenes from the Soft Coral *Klyxum simplex*

Shwu-Li Wu,[1,2] Jui-Hsin Su,[3,4] Chiung-Yao Huang,[1] Chi-Jen Tai,[1] Ping-Jyun Sung,[3,4] Chih-Chung Liaw,[1] and Jyh-Horng Sheu*[1,5]

[1] Department of Marine Biotechnology and Resources, National Sun Yat-sen University, Kaohsiung 804, Taiwan

[2] Center of General Studies, National Kaohsiung Marine University, Kaohsiung 811, Taiwan

[3] National Museum of Marine Biology & Aquarium, Pingtung 944, Taiwan;

[4] Graduate Institute of Marine Biotechnology, National Dong Hwa University, Pingtung 944, Taiwan

[5] Division of Marine Biotechnology, Asia-Pacific Ocean Research Center, National Sun Yat-sen University, Kaohsiung 804, Taiwan

*To whom correspondence should be addressed. Tel.: 886-7-5252000 ext. 5030, Fax: 886-7-5255020. E-mail: sheu@mail.nsysu.edu.tw

For compound 1:

Figure S1-1. ^{1}H NMR spectrum (500 MHz) of compound **1** in $CDCl_3$.

Figure S1-2. ^{13}C NMR spectrum (125 MHz) of compound **1** in $CDCl_3$.

For compound 2:

Figure S2-1. ^{1}H NMR spectrum (500 MHz) of compound **2** in $CDCl_3$.

Figure S2-2. ^{13}C NMR spectrum (125 MHz) of compound **2** in $CDCl_3$.

For compound 3:

Figure S3-1. ^{1}H NMR spectrum (500 MHz) of compound **3** in $CDCl_3$.

Figure S3-2. ^{13}C NMR spectrum (125 MHz) of compound **3** in $CDCl_3$.

For compound 4:

Figure S4-1. ^{1}H NMR spectrum (500 MHz) of compound **4** in $CDCl_3$.

Figure S4-2. ^{13}C NMR spectrum (125 MHz) of compound **4** in $CDCl_3$.

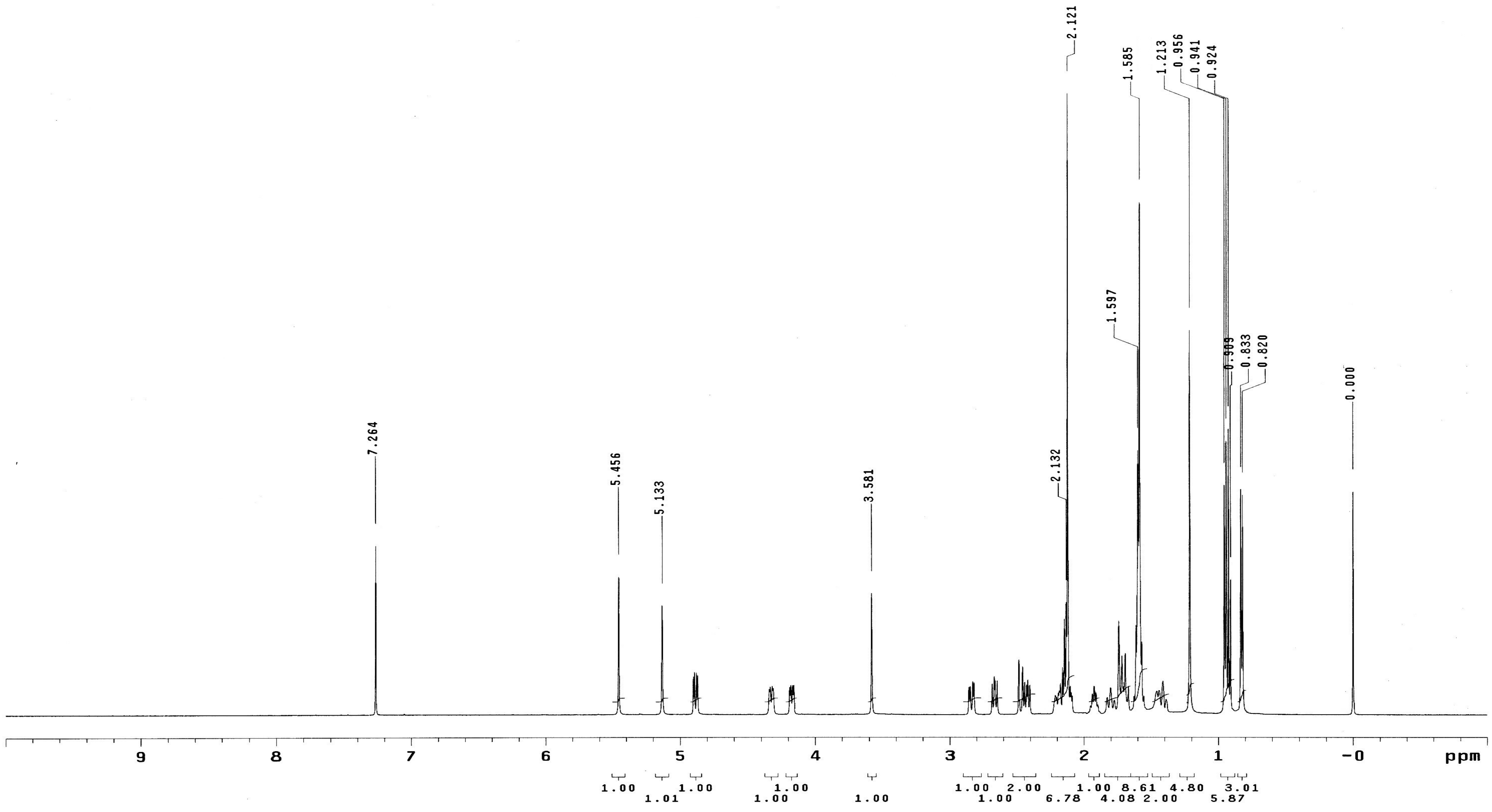

Figure S1-1. ^{1}H NMR spectrum (500 MHz) of compound **1** in $CDCl_3$.

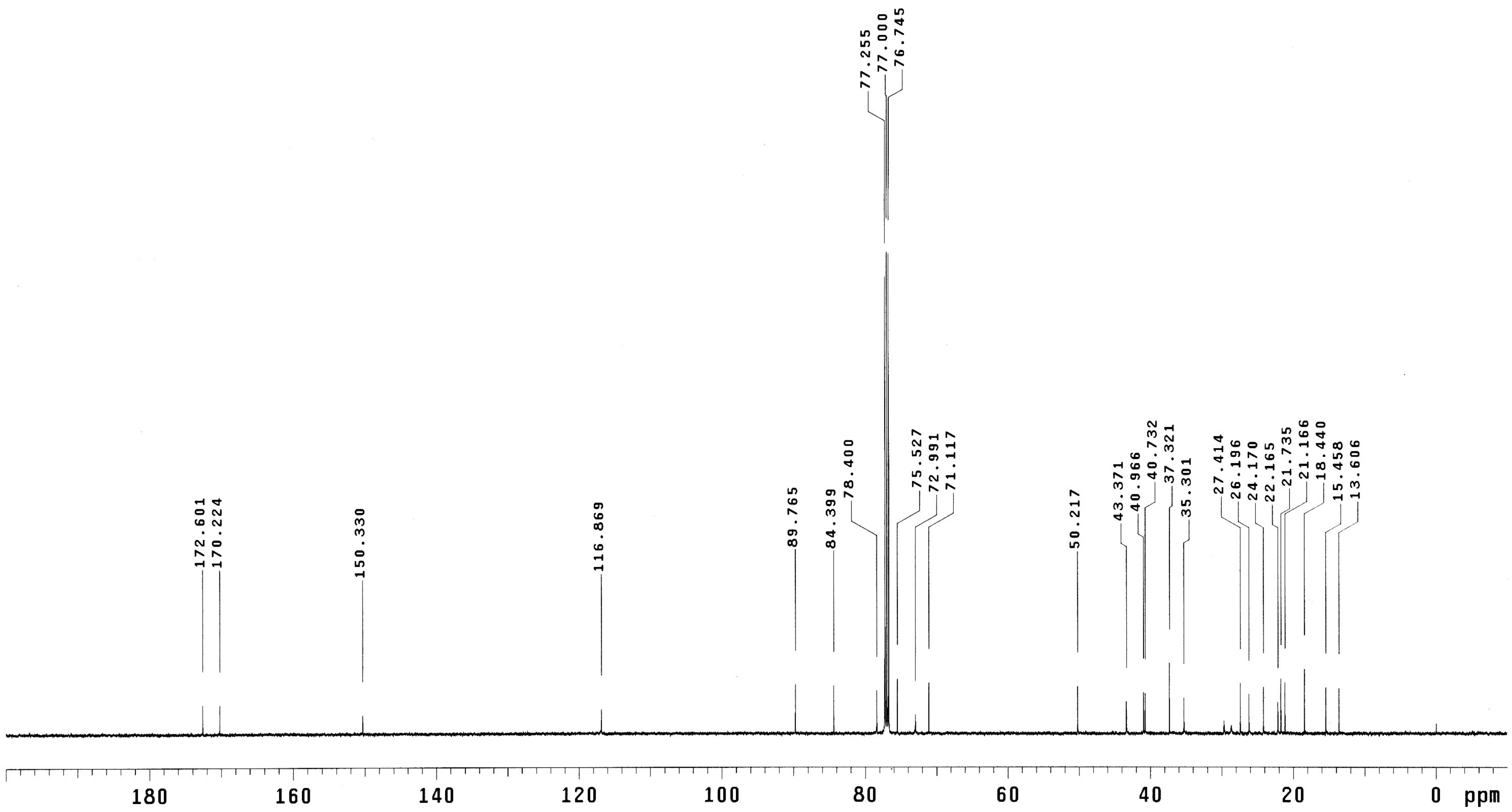

Figure S1-2. ^{13}C NMR spectrum (125 MHz) of compound **1** in $CDCl_3$.

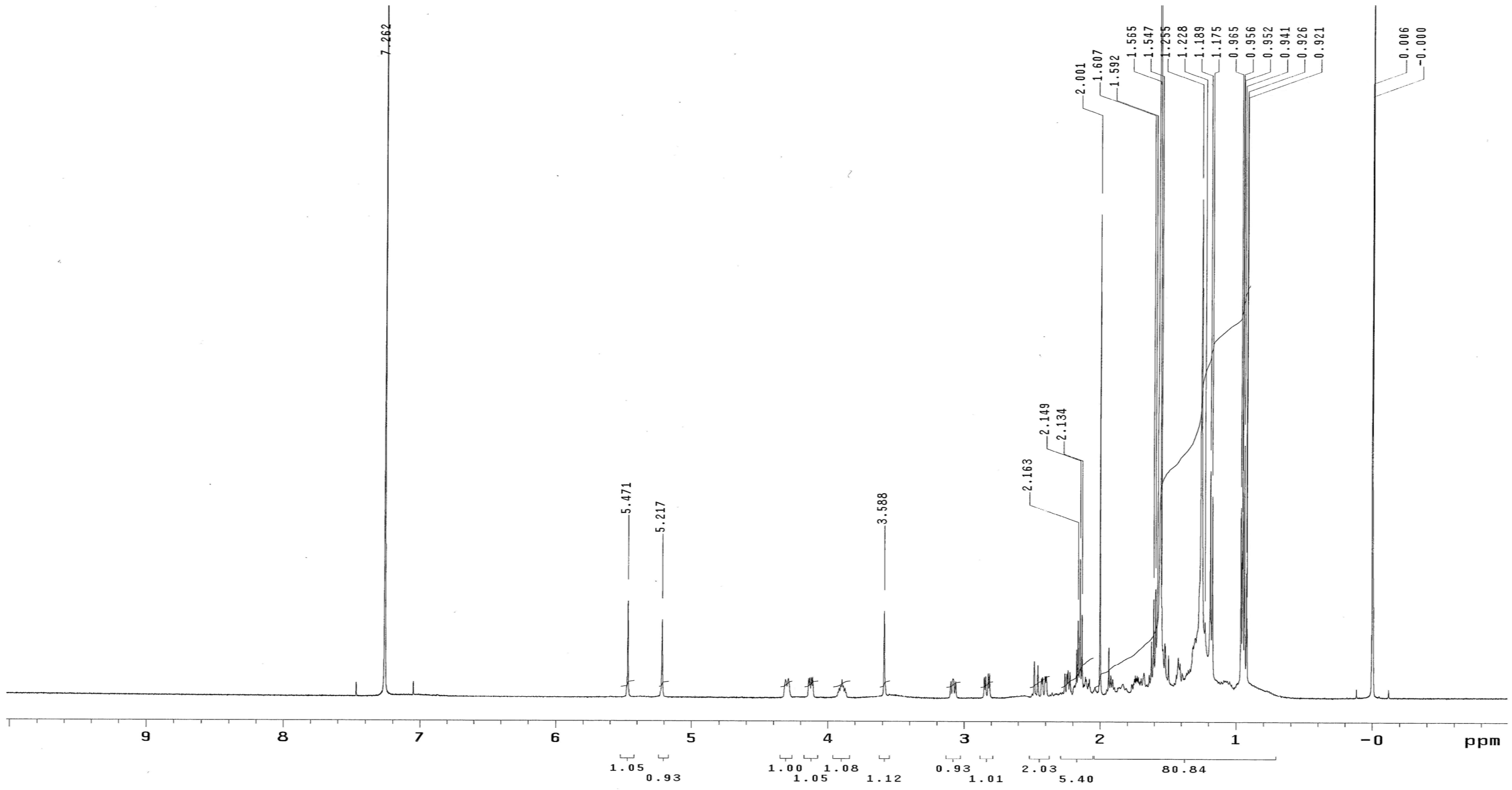

Figure S2-1. ^{1}H NMR spectrum (500 MHz) of compound **2** in $CDCl_3$.

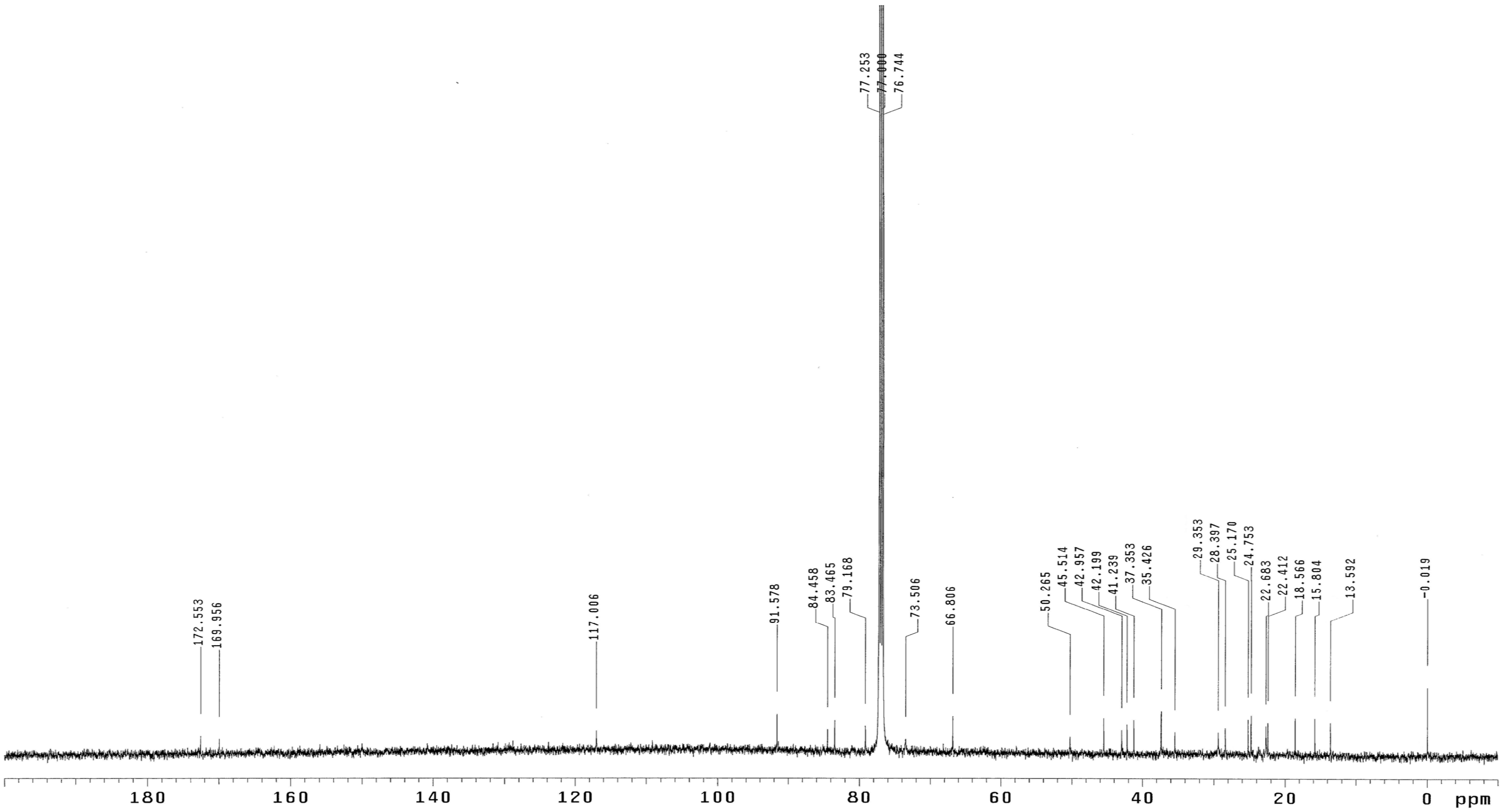

Figure S2-2. ^{13}C NMR spectrum (125 MHz) of compound **2** in $CDCl_3$.

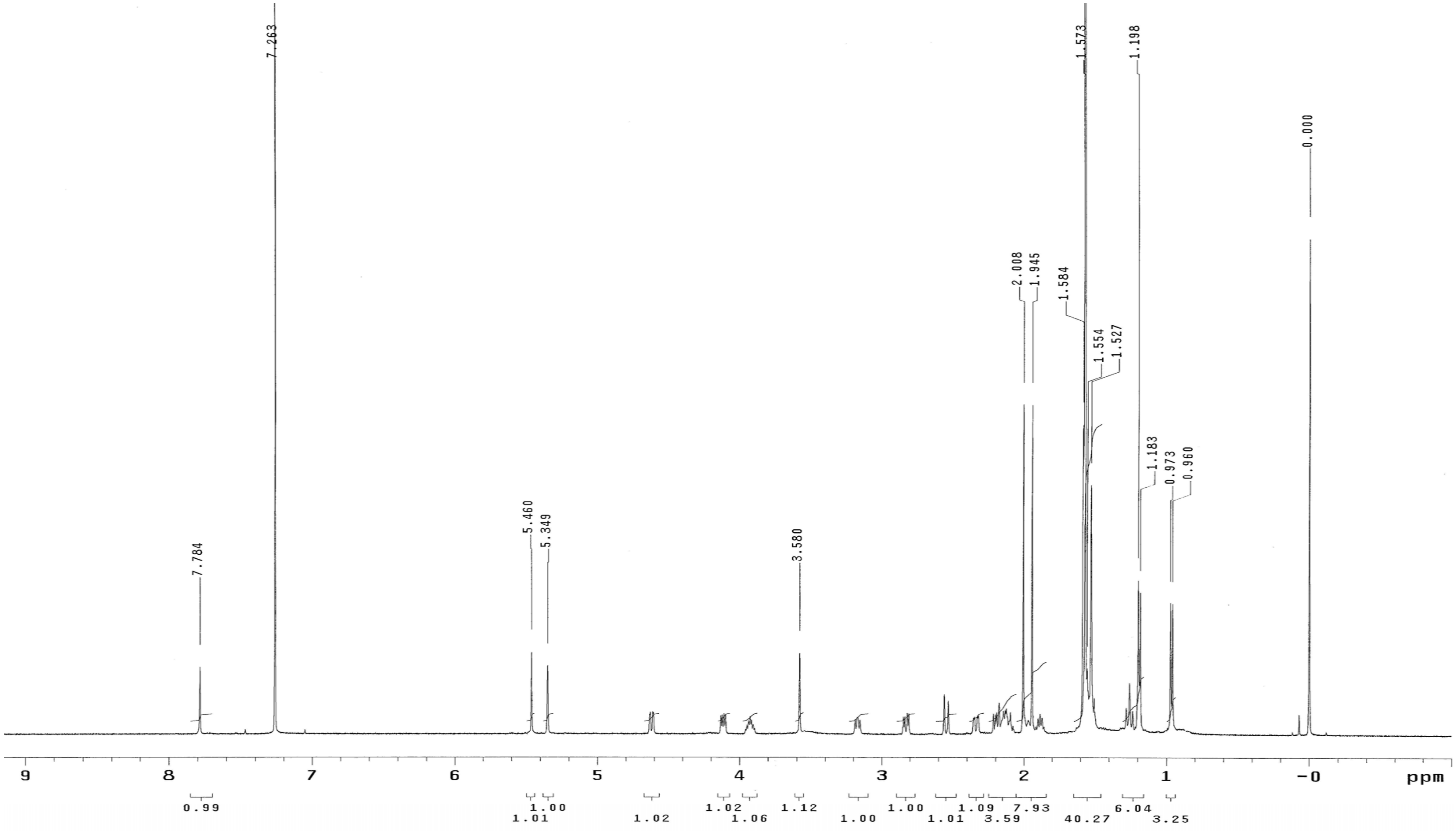

Figure S3-1. ^{1}H NMR spectrum (500 MHz) of compound **3** in $CDCl_3$.

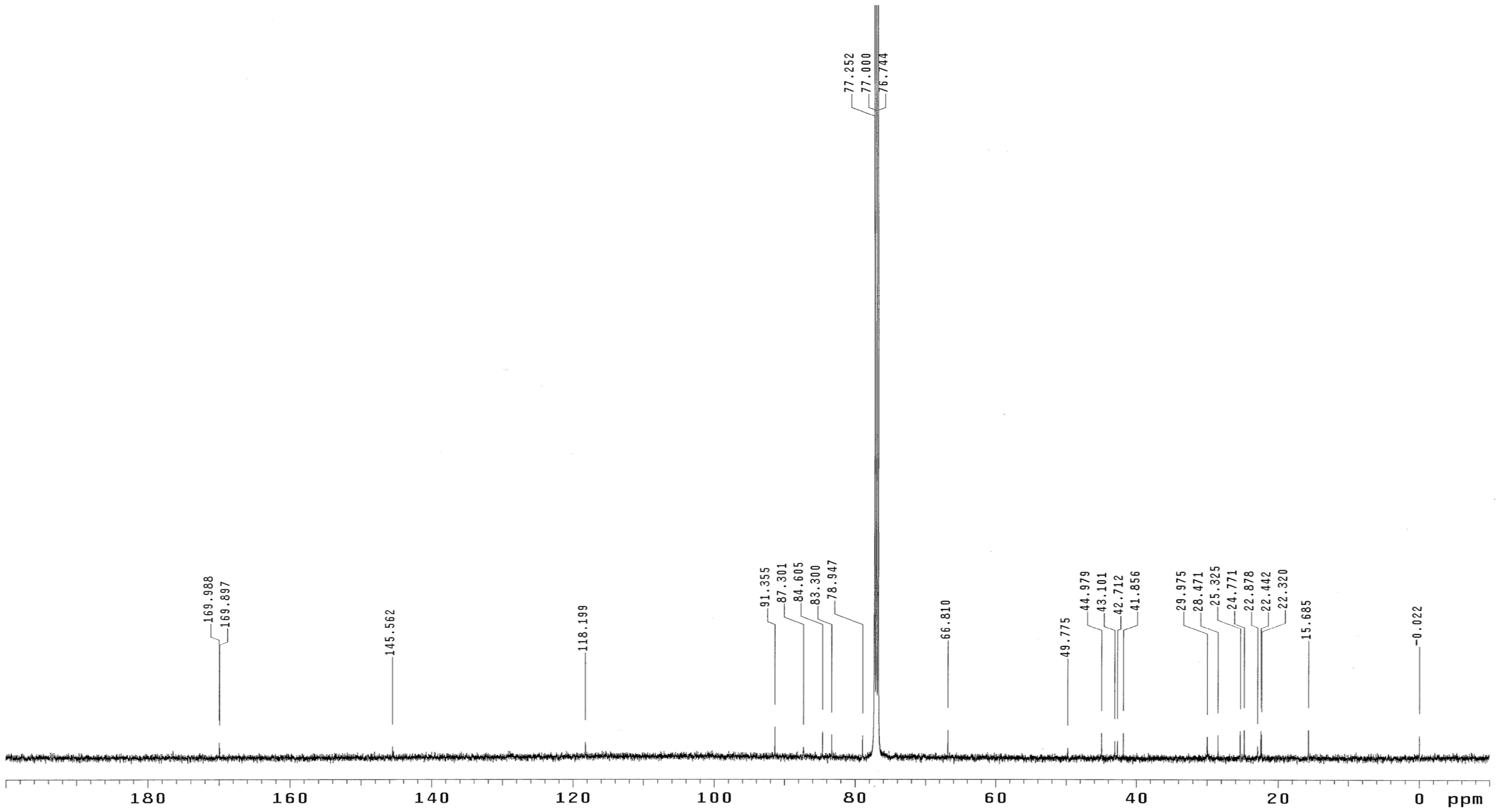

Figure S3-2. ^{13}C NMR spectrum (125 MHz) of compound **3** in $CDCl_3$.

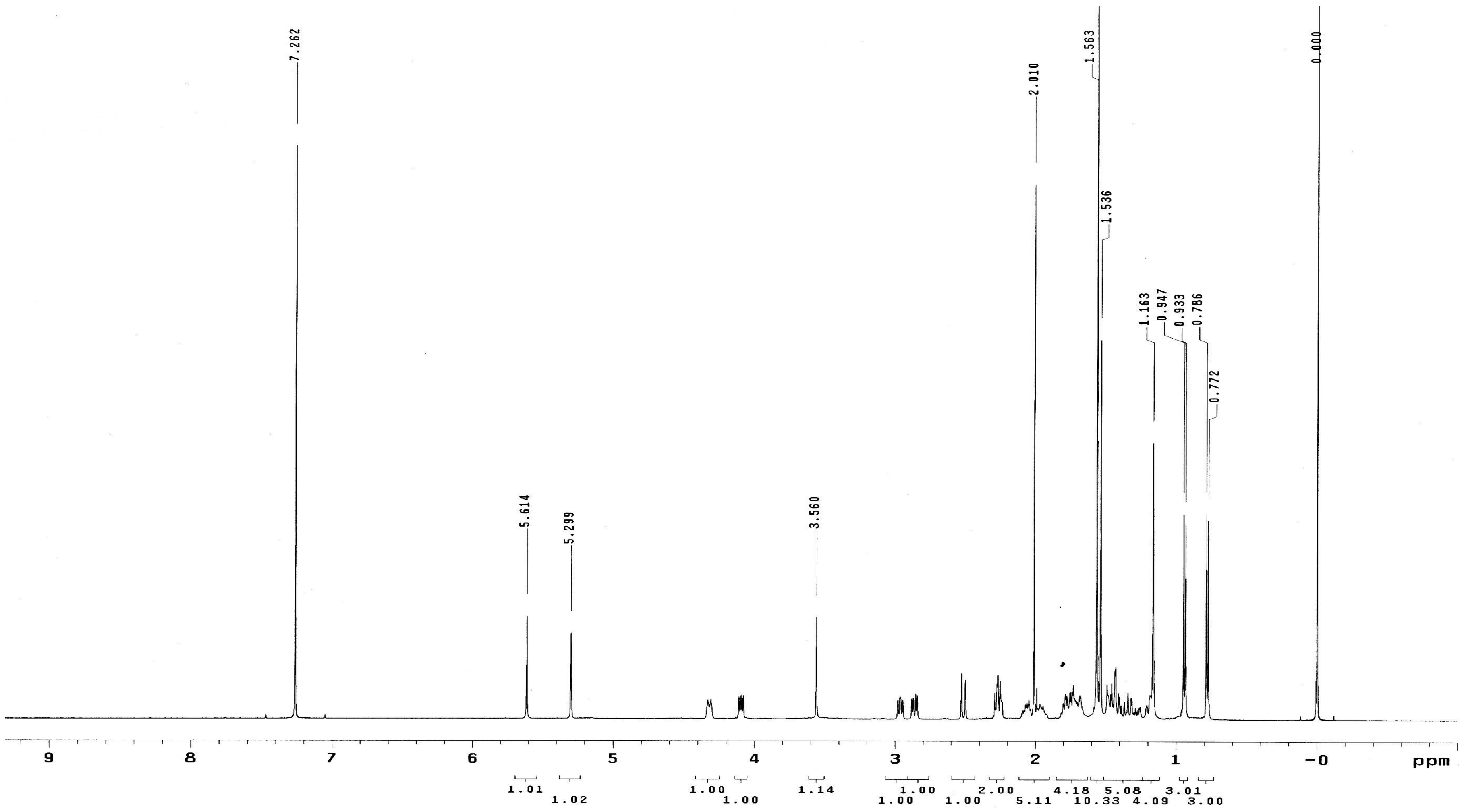

Figure S4-1. ^{1}H NMR spectrum (500 MHz) of compound **4** in $CDCl_3$.

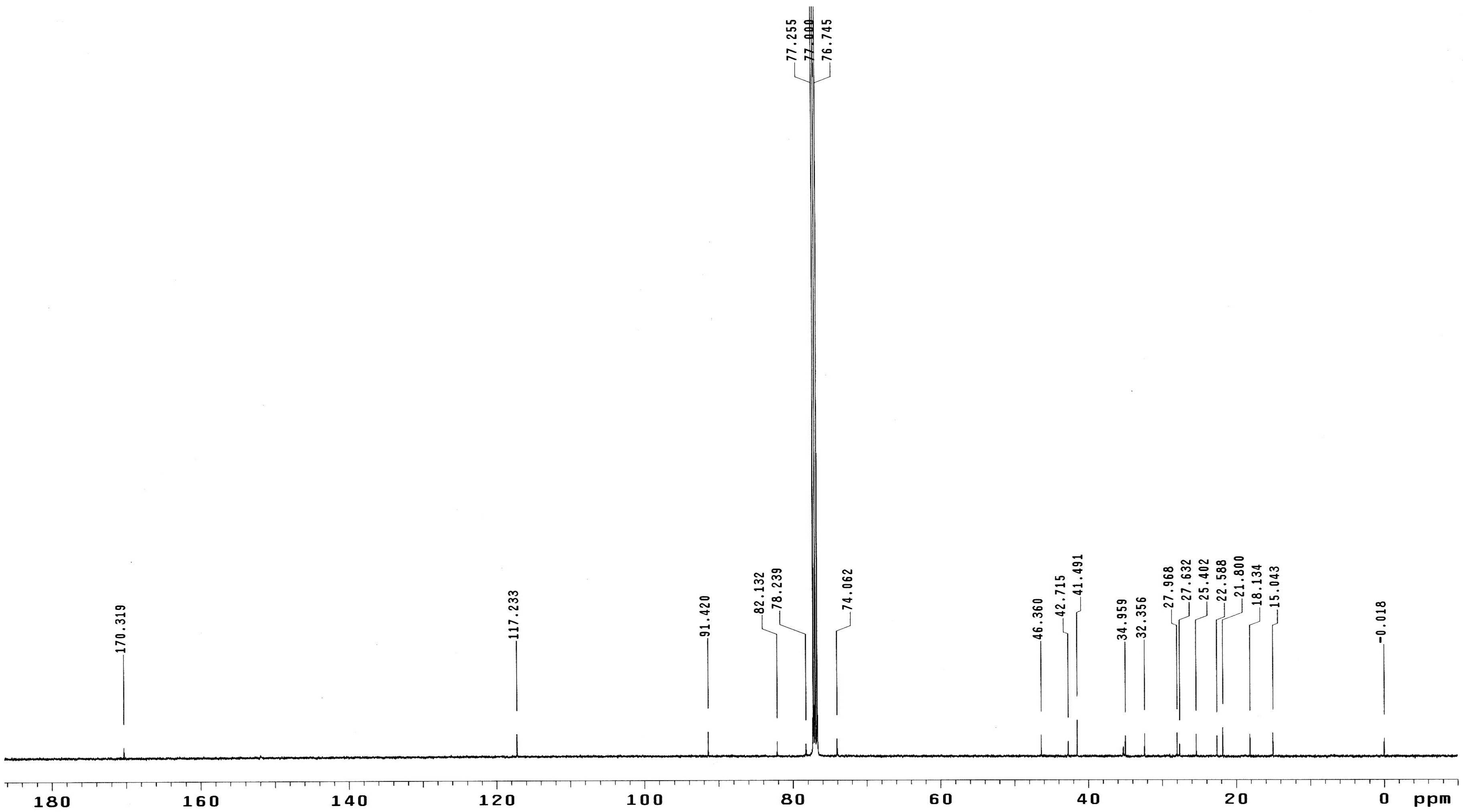

Figure S4-2. ^{13}C NMR spectrum (125 MHz) of compound **4** in $CDCl_3$.
